# A Rare Case of Inadvertent Iatrogenic Osmotic Demyelination Syndrome

**DOI:** 10.7759/cureus.16180

**Published:** 2021-07-04

**Authors:** Kasopefoluwa O Akinbamijo, Ifeoluwa R Malmberg, Candace Griffith, Oluwatomisin Aluko, Rajesh Thirumaran

**Affiliations:** 1 Internal Medicine, Mercy Catholic Medical Center, Darby, USA; 2 Internal Medicine, Mercy Hospital, Yeadon, USA; 3 Hematology/Oncology, Mercy Catholic Medical Center, Darby, USA

**Keywords:** osmotic demyelination syndrome, central pontine myelinolysis, hyperglycemic hyperosmolar non-ketotic syndrome, diabetic keto acidosis, extrapontine myelinolysis

## Abstract

A 73-year-old African American male presented with altered mental status, severe hyperglycemia, and acute kidney injury. His metabolic derangements including hyperglycemia and hyponatremia were initially thought to be the cause of his encephalopathy. While managing his hyperglycemic hyperosmolar non-ketotic state, he received intravenous rehydration with almost three times his physiologic requirement for normalization of his electrolyte abnormalities. After the correction of the metabolic derangements, he remained confused with dysarthria and labile mood. Magnetic resonance imaging of the brain revealed osmotic demyelination syndrome.

## Introduction

Osmotic demyelination syndrome (ODS) as an umbrella term for central pontine myelinolysis (CPM) and extrapontine myelinolysis is underutilized but underlines the pathophysiology of the destruction of the myelin fibers throughout the neural structures [1]. Typical findings in ODS include dysarthria, dysphagia, paraparesis, seizures, confusion, disorientation, obtundation, and coma. We also find the phenomenon known as locked-in syndrome when ODS occurs and the patients are unable to move or speak but are fully aware of all that is going on around them. For patients with diabetic ketoacidosis (DKA)/hyperglycemic hyperosmolar non-ketotic state (HHNK) as the underlying pathology, proper glycemic control as well as dietary modification and medication adherence are key to treatment as well as follow-up with glycated hemoglobin after discharge [2].

Here we describe a case of development of ODS secondary to iatrogenic over-rehydration in the course of treatment of hyperglycemia, a rare occurrence but not an isolated case.

## Case presentation

We present the case of a 72-year-old male, with a history of diabetes, hypertension, and hyperlipidemia, who was found by his neighbor and brought to the emergency department for being confused and appearing ill. He was last seen normal 36 hours prior by his aide. He was found to be incoherent, dehydrated, tachycardic, hypothermic (94.6 degrees Fahrenheit), and hypotensive (79/50 mmHg). Physical examination revealed an elderly patient, only oriented to person and unable to describe the onset of symptoms. Glasgow coma scale was noted to be 14. His speech was slurred and garbled with dry mucous membranes. His National Institute of Health Stroke Scale was noted to be 3. Laboratory results revealed severe metabolic acidosis; see Table 1 for values on admission.

**Table 1:** Laboratory values on admission

Lab	Value on admission	Normal range
pH	7.25	7.35-7.45
Bicarb (HCO_3_)	21.3 mmol/L	23-29 mmol/L
Lactate	5.2 mmol/L	0.5-1.9 mmol/L
Creatinine	4.8 mg/dL	0.6-1.3 mg/dL
Glucose	897 mg/dL	70-110 mg/dL
Sodium	126 mEq/L	136-145 mEq/L
Anion Gap	27	6-15
Leukocytosis	17k/µL	4.5-11k/µL
Betahydroxybutyrate	4.40 mmol/L	0.02-0.27 mmol/L

He had presented with multiple metabolic derangements including hyperglycemia-related hyponatremia 126 mEq/L (normal range: 136-145 mEq/L), elevated phosphorus, magnesium, and creatine kinase, and an AKI with creatinine >4 mg/dL (normal range: 0.6-1.3 mg/dL). He received approximately 13 L of fluid in-house. He was resuscitated with 6 L of dextrose saline and 2.5 L of lactated Ringers in the first 48 h. He received another 3 L over the next 48 h. He was started on an insulin drip and admitted to the intensive care unit (ICU) with the initial diagnosis of HHNK, AKI, and septic shock, briefly requiring peripheral vasopressors. He received warmed intravenous fluids for resuscitation, a warming blanket was utilized, and a Foley catheter was placed for possible urinary obstruction.

The initial computed tomography (CT) scan of his brain showed no intracranial hemorrhage or acute infarct. He spent two days in the ICU, during which time his blood sugars normalized, he was transitioned to subcutaneously administered insulin, and AKI started to resolve. He was still noted to have moderate dysarthria with inappropriate emotional outbursts and persistent confusion despite ruling out acute infectious and metabolic encephalopathy as possible differentials.

**Figure 1:**
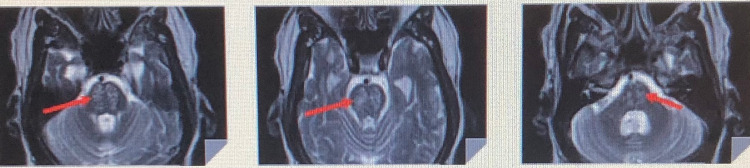
Patient magnetic resonance imaging showing findings leading up to diagnosis Red arrows showing the patchy diffusion restriction with associated T2/fluid-attenuated inversion recovery.

Neurology team was consulted six days into the admission and recommended obtaining a magnetic resonance imaging (MRI) with angiography. MRI revealed patchy diffusion restriction with associated T2/fluid-attenuated inversion recovery abnormal signal within the pons, a finding often seen in ODS. Therefore, the diagnosis was made in our patient (Figure 1).

In our patient, his encephalopathy and confusion resolved, as did the inappropriate emotional outbursts. On discharge 10 days later, all electrolytes normalized including sodium and glucose. It was recommended that he follow up with neurology as an outpatient, and obtain a repeat MRI to reassess the lesion.

## Discussion

ODS often occurs linked to rapid correction of hyponatremia, although in our patient, given his hyperglycemic state, initial glucose of 897, his sodium of 126 mEq/L was recalculated and corrected to 145 mEq/L. Our clinical assessment of ODS is usually in the setting of rapid correction of hyponatremia, especially in patients presenting with sodium levels less than or equal to 105 mEq/L (normal range: 136-145 mEq/L). It is rarely documented in DKA/HHNK; however, imaging showed the phenomenon in this case.

As described in the literature by Hseih et al., over-hydration of patients while important in the treatment of DKA/HHNK can tip patients over into development of ODS [3]. While patients are usually dehydrated, we must ensure that we keep an eye on the electrolyte imbalances and attempt to minimize rapid shifts. We must also be aware of the early changes that may be pointers to ODS. Early identification may lead to a full or at best partial recovery, which could take some time [4].The development of ODS in this patient supports the work put forth by Kusumoto et al., highlighting the need to monitor closely how we fluid resuscitate patients who are in hyperglycemic and hyperosmotic states [5].

MRI is usually instrumental in the diagnosis of ODS when suspected, and CT scans can be utilized but are not reliable. The findings of patchy diffusion restriction within the pons on the patient’s MRI prompted discussions with neurology and nephrology about the possibility of ODS developing secondary to metabolic derangements and rapid fluid shifts in hyperglycemic patients, seen in a case report by Saini et al. [6]. Our case report corroborates the occurrence of CPM in patients with hyperglycemic DKA/HHNK despite appropriate sodium corrections [7,8].

Management usually involves intensive supportive therapies paired with correcting the electrolyte derangements. Given the pseudobulbar effect noticed in our patient, dextromethorphan/quinidine was added on to his regimen by neurology as was noted in the study by Rosen [9]. 

## Conclusions

ODS is a well-known phenomenon and one we actively guard against when a patient with hyponatremia is admitted and we begin correction. Scheduled monitoring of electrolytes and constant fluid adjustments are a mainstay. 

While the goal is always to normalize the glucose levels and rehydrate in the initial management of DKA and HHNK, caution and vigilance must be exercised to avoid maleficence secondary to rapid correction of electrolytes, especially sodium. Patient’s full recovery while not guaranteed is possible when ODS develops if treatment is initiated expediently. 
